# Diagnostic Usefulness of SP-D, CCL2/MCP-1, and IL-18 in Assessing Respiratory Function and Risk of Pulmonary Fibrosis in COVID-19 Patients

**DOI:** 10.3390/ijms27104190

**Published:** 2026-05-08

**Authors:** Bogdan Cylwik, Kacper Gan, Marcin Kazberuk, Ewa Gruszewska, Anatol Panasiuk, Magdalena Sienkiewicz, Malgorzata Wojtkowska, Lech Chrostek

**Affiliations:** 1 Department of Paediatric Laboratory Diagnostics, Medical University of Bialystok, 15-269 Bialystok, Poland; 2 Department of Gastroenterology, Hepatology and Internal Diseases, Voivodeship Hospital in Bialystok, 15-278 Bialystok, Poland; kaspergan@gmail.com (K.G.); anatol.panasiuk@panasiuk.pl (A.P.); 3 Department of Biochemical Diagnostics, Medical University of Bialystok, 15-269 Bialystok, Poland; ewa.gruszewska@umb.edu.pl (E.G.); lech.chrostek@umb.edu.pl (L.C.); 4 Department of Clinical Medicine, Medical University of Bialystok, 15-269 Bialystok, Poland; 5 Department of Paediatric Laboratory Diagnostics, Medical University of Bialystok Children’s Clinical Hospital, 15-274 Bialystok, Poland; magdalena.sienkiewicz@udsk.pl (M.S.); malgorzata.wojtkowska@udsk.pl (M.W.)

**Keywords:** SP-D, CCL2/MCP-1 IL-18, COVID-19 patients

## Abstract

This study evaluated the diagnostic usefulness of surfactant protein D (SP-D), chemokine C-C motif ligand 2/monocyte chemoattractant protein-1 (CCL2/MCP-1), and interleukin 18 (IL-18) in patients with SARS-CoV-2 infection. The authors focused on lung failure assessment, lung parenchyma involvement, and the early assessment of the risk of developing pulmonary fibrosis at the onset of COVID-19. The study group included 87 patients with COVID-19 and 45 healthy subjects. Concentrations of all three markers were measured using the enzyme immunoassay method. The serum SP-D, CCL2/MCP-1, and IL-18 concentrations were significantly higher in the COVID-19 patients before admission to the hospital than those in the controls (*p* < 0.001 for all comparisons). Differences were only found in the IL-18 levels between the groups categorized according to disease severity (*p* < 0.001); levels were significantly higher in patients with critical disease severity compared to those with moderate disease severity (*p* < 0.001). IL-18 also showed a positive correlation with the disease severity (*p* = 0.025). SP-D and IL-18 levels varied depending on the amount of oxygen administration (*p* = 0.017 and *p* < 0.001, respectively). Among the blood gas parameters, SP-D levels were negatively associated with the partial pressure of arterial oxygen (PaO_2_) and oxygen saturation (O_2_Sat) (*p* = 0.026 and *p* = 0.048, respectively), IL-18 with O_2_Sat (*p* = 0.036), and CCL2/MCP-1 with PaO_2_ (*p* = 0.042). SP-D and IL-18 were significantly negatively correlated with oxygen therapy (*p* = 0.003 and *p* = 0.014, respectively). Conversely, CCL2/MCP-1 was significantly positively correlated with the pulmonary involvement severity (*p* = 0.017) and level of hyaluronic acid (HA), a marker of fibrosis (*p* = 0.042). We also observed a significant correlation between the IL-18 level and the pulmonary involvement severity (*p* < 0.001), HA (*p* < 0.001), and other markers of fibrosis. In summary, our study’s results indicate that SP-D, CCL2/MCP-1, and IL-18 may be used to assess lung function in the early stage of COVID-19 infection; additionally, SP-D may serve as an indicator of alveolar injury, while CCL2/MCP-1 and IL-18 may be markers of lung parenchyma involvement and potential predictors of the development of pulmonary fibrosis.

## 1. Introduction

Coronavirus disease (COVID-19) is a highly infectious illness that emerged abruptly in December 2019 and became a global pandemic by March 2020, affecting populations worldwide [1,2]. By December 2025, there were about 779 million confirmed cases and around 7.1 million deaths had been linked to the disease [3]. COVID-19 is an illness caused by severe acute respiratory syndrome coronavirus 2 (SARS-CoV-2). Most cases of this disease are mild or moderate and do not require hospitalization or advanced medical care. Notwithstanding the above, a significant subset of patients become severely ill. A small number of patients, particularly those with severe or critical disease, develop pulmonary manifestations such as pneumonia and pulmonary edema. These can progress to acute respiratory distress syndrome (ARDS) or multi-organ failure, both linked to poor outcomes and high mortality rates. Hence, the long-term course of COVID-19 infection remains a subject of intensive study [4]. One of the most serious long-term complications of post-COVID-19 is pulmonary fibrosis. This condition is characterized by chronic progressive scarring of lung tissue, leading to impaired lung function and breathlessness [5]. It can develop particularly in patients who had severe COVID-19 pneumonia or acute respiratory distress syndrome (ARDS) or in those who required intensive respiratory support, such as mechanical ventilation. Pulmonary fibrosis occurs due to interstitial collagen deposition and alveolar remodeling following severe lung injury caused by SARS-CoV-2 infection [6,7]. Risk factors include advanced age, male sex, and the severity of the initial COVID-19 illness [6,7,8,9].

Lung failure and the potential risk of developing pulmonary fibrosis are diagnosed using a combination of clinical assessment, pulmonary function tests, serum laboratory tests, and CT imaging studies (CTSS) [6,8]. Pulmonary function tests include forced vital capacity (FVC), forced expiratory volume in 1 s (FEV1), the FEV1/FVC ratio, total lung capacity (TLC), residual volume (RV), and diffusion capacity for carbon monoxide (DLCO) [10]. Laboratory tests, especially arterial blood gas (ABG) and CO-oximetry, are performed in the laboratory and at the patient’s bedside as point-of-care testing (POCT). Furthermore, imaging studies, such as CTSS (chest computed tomography severity score), allow for the evaluation of the degree of pulmonary involvement and possible lung parenchyma damage in SARS-CoV-2 infection. As for laboratory tests, some serum biomarkers correlate with the severity of pulmonary fibrosis, but specifically in long-term post-COVID-19 complications [11,12,13,14,15,16,17]. These include KL-6 (Krebs von den Lungen-6), MMP-8 (matrix metalloproteinase-8), IL-10 (interleukin-10), and ACE2 (human angiotensin-converting enzyme 2). However, no specific laboratory marker has been established for evaluating the risk of COVID-19 pulmonary fibrosis. Therefore, it is necessary to identify potential biomarkers that can predict the progression of pulmonary fibrosis or other adverse consequences.

In our study, we examined the diagnostic usefulness of three other laboratory tests, namely, SP-D (surfactant protein D), CCL2/MCP-1 (chemokine C-C motif ligand 2/monocyte chemoattractant protein 1), and IL-18 (interleukin 18). Our objective was to assess lung function, the degree of lung parenchyma involvement, as well as potential development of pulmonary fibrosis at the onset of COVID-19. SP-D is a pulmonary surfactant protein located within the alveolar space. Recently, it was shown that SP-D levels significantly increased in both patients with ARDS and those with fibrosis-like changes on CT scans. These findings suggest that most patients with COVID-19-induced ARDS have a higher risk of pulmonary fibrosis than patients with ARDS [17]. CCL2/MCP-1 is a chemokine that regulates cellular processes and recruits monocytes, macrophages, and dendritic cells to sites of inflammation (especially in the lungs), produced in response to tissue injury or infection, including SARS-CoV-2 infection [18]. CCL2 plays a key role in both the acute inflammatory phase and the development of post-COVID-19 pulmonary fibrosis. This occurs through fostering monocyte/macrophage infiltration, stimulating fibroblast collagen synthesis, and exacerbating lung tissue damage [19]. Furthermore, IL-18 levels have been reported to be significantly elevated and correlated with severe disease, contributing to inflammatory lung injury and potentially to the development of pulmonary fibrosis as a post-COVID-19 complication [20,21,22].

## 2. Results

Table 1 presents the outcomes of standard laboratory assessments for the COVID-19 patients and control subjects. Among the biochemical findings, median serum activities of ALT, AST, GGT, and LDH were markedly higher at hospital admission than in controls (for all comparisons, *p* < 0.001). Following hospitalization, only AST and LDH decreased significantly (*p* = 0.005 and *p* < 0.001, respectively). All inflammatory markers: IL-6, CRP, procalcitonin, ferritin, and fibrinogen were significantly elevated in patients prior to admission versus the controls (for all comparisons, *p* < 0.001). After hospitalization, CRP dropped markedly (*p* < 0.001), while ferritin increased (*p* = 0.005). Levels of IL-6, procalcitonin, and fibrinogen showed no significant change. Regarding the hematological parameters, COVID-19 patients prior to hospitalization exhibited lower Hb, Ht, and RBC, and higher MCV compared to the healthy controls (*p* < 0.001, *p* = 0.028, *p* < 0.001, and *p* = 0.001, respectively). Following admission, MCV and platelet counts rose (*p* = 0.041 and *p* = 0.002), with white blood cell counts declining (*p* = 0.047). Blood gas analysis indicated elevated pH and BE, alongside reduced PaO_2_ and PaCO_2_, in SARS-CoV-2 patients before hospitalization relative to the controls (*p* < 0.001, *p* = 0.002, *p* = 0.008, and *p* = 0.002). O_2_Sat was lower in patients though the difference did not reach statistical significance.

**Figure 1:**
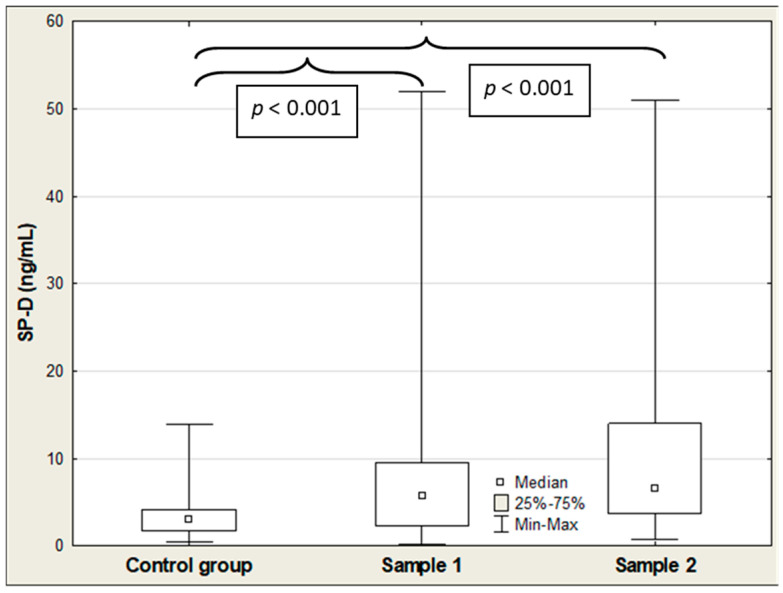
SP-D levels in the control group compared to COVID-19 patients at admission (Sample 1) and post-hospitalization (Sample 2).

**Figure 2:**
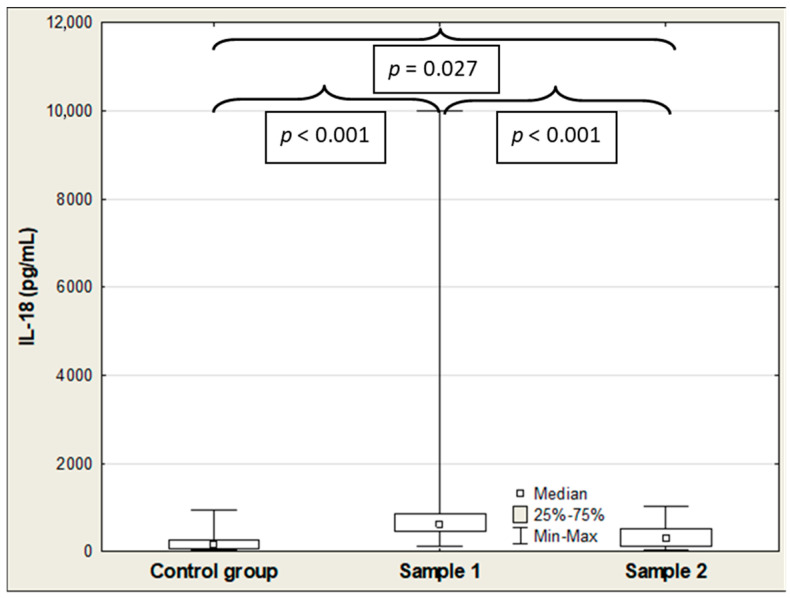
IL-18 concentrations in the control group and COVID-19 patients at admission (Sample 1) and after hospitalization (Sample 2).

**Figure 3:**
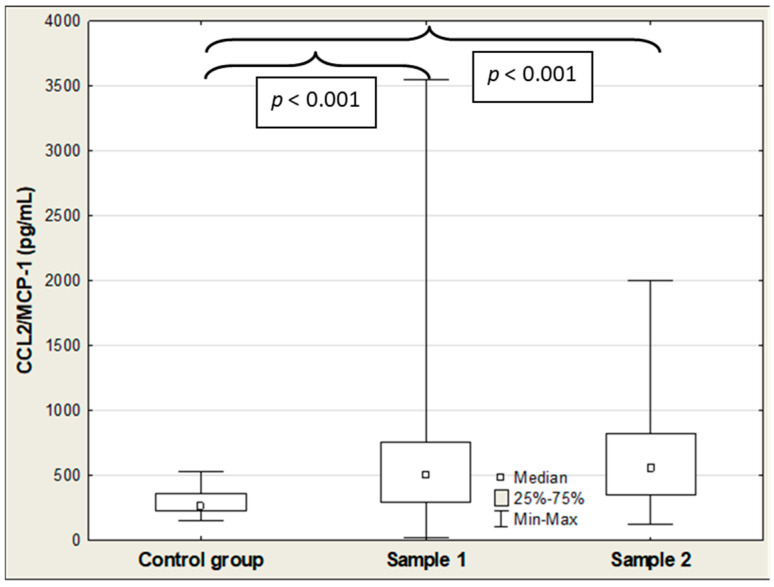
CCL2/MCP-1 levels in the healthy controls and COVID-19 patients at admission (Sample 1) versus post-hospitalization (Sample 2).

Table 2 shows the results of SP-D, IL-18, and CCL2/MCP-1 in COVID-19 patients and controls. The median serum SP-D, CCL2/MCP-1, and IL-18 concentrations were significantly elevated in the COVID-19 patients on admission (Sample 1) compared to the control group (5.73 vs. 3.04 ng/mL (*p* < 0.001); 606 vs. 159 pg/mL (*p* < 0.001); and 504 vs. 262 pg/mL (*p* < 0.001), respectively), and the values of these parameters remained significantly higher in the patient group compared to the healthy individuals after hospitalization (Sample 2) (6.53 vs. 3.04 ng/mL (*p* < 0.001); 302 vs. 159 pg/mL (*p* = 0.027); and 553 vs. 262 pg/mL (*p* < 0.001), respectively) (Table 2; Figure 1, Figure 2 and Figure 3). Among the three parameters, only the median IL-18 level was significantly lower in the patients after hospitalization compared to admission levels before treatment (606 vs. 302 pg/mL (*p* < 0.001)) (Table 2; Figure 2). All Figures display results as medians with interquartile range (Q1–Q3), and as a maximum and minimum values, additionally (large error bars in the graph show a wide dispersion of the raw data).

Table 3 presents the results of SP-D, IL-18, and CCL2/MCP-1 in different patient groups according to the cytokine storm, comorbidities, vaccination, and those surviving/non-surviving. Patients with cytokine storm showed elevated serum levels of SP-D, CCL2/MCP-1, and IL-18 compared to those without, though only the IL-18 difference reached statistical significance (590 vs. 517 pg/mL; *p* < 0.001). Likewise, these biomarkers were higher in survivors than non-survivors, with significance limited to IL-18 (878 vs. 578 pg/mL; *p* < 0.001). No statistically significant differences in SP-D, CCL2/MCP-1, or IL-18 levels were observed between patients with coexisting chronic conditions and those without. Patients with prior SARS-CoV-2 vaccination revealed higher median levels of these markers than unvaccinated individuals, though the differences did not reach statistical significance.

Table 4 presents the relationships between SP-D, IL-18, and CCL2/MCP-1 levels and various laboratory parameters in patients with COVID-19. For liver function markers, serum IL-18 exhibited positive correlations with ALT (R = 0.298; *p* = 0.003), AST (R = 0.389; *p* < 0.001), GGT (R = 0.242; *p* = 0.019), and total bilirubin (R = 0.330; *p* = 0.001). IL-18 also positively correlated with LDH (R = 0.398; *p* < 0.001). Regarding inflammation markers, IL-18 was positively associated with IL-6 (R = 0.508; *p* < 0.001), CRP (R = 0.445; *p* < 0.001), procalcitonin (R = 0.483; *p* < 0.001), and ferritin (R = 0.544; *p* < 0.001). Both CCL2/MCP-1 and IL-18 exhibited positive correlations with IFN-γ (R = 0.265; *p* = 0.009 and R = 0.508; *p* < 0.001, respectively). In terms of the fibrosis markers, both CCL2/MCP-1 and IL-18 showed a significant positive association with HA (R = 0.218 (*p* = 0.042) and R = 0.340 (*p* < 0.001), respectively). Meanwhile, only IL-18 was positively correlated with TNF-α (R = 0.269; *p* = 0.008) and galectin-3 (R = 0.547; *p* < 0.001). Among the hematological parameters, IL-18 was positively correlated with WBC (R = 0.326; *p* = 0.001). With regard to the blood gas parameters, SP-D levels were negatively associated with PaO_2_ and O_2_Sat (R = −0.240 (*p* = 0.026) and R = −0.206 (*p* = 0.048), respectively), IL-18 with O_2_Sat (R = −0.218 (*p* = 0.036)), and CCL2/MCP-1 with PaO_2_ (R = −0.219 (*p* = 0.042)). There is a negative association between SP-D and CCL2/MCP-1 and PaO_2_ (these parameters increase while PaO_2_ tends to decrease). It usually points to worsening lung injury or inflammation, with SP-D showing a stronger relationship. Similarly, a negative correlation was observed between SP-D, IL-18, and O_2_ saturation, with the strength of the association being equal for both parameters.

**Figure 4:**
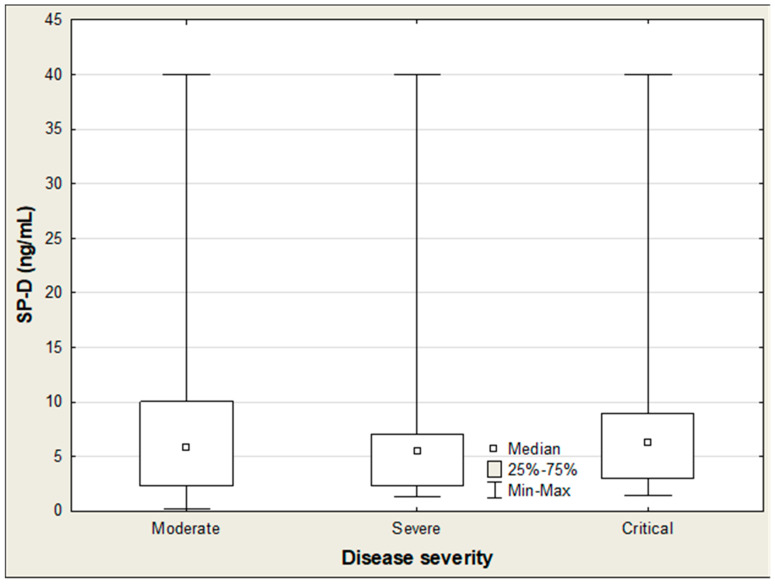
SP-D levels in COVID-19 patients according to disease severity.

**Figure 5:**
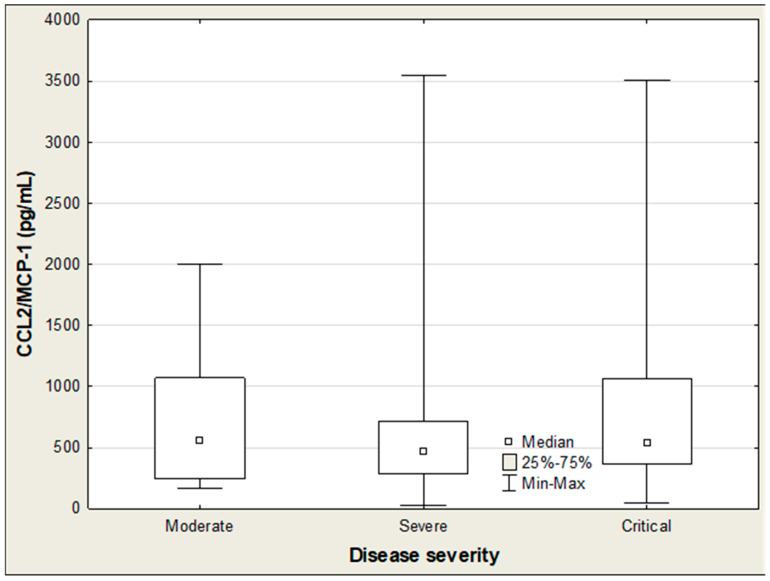
CCL2/MCP-1 levels in COVID-19 patients according to disease severity.

**Figure 6:**
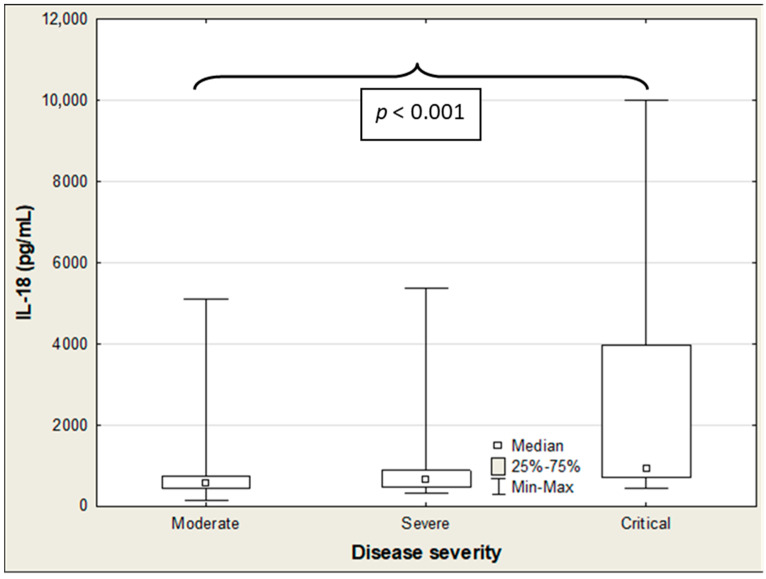
IL-18 concentrations in the COVID-19 patients according to disease severity.

Table 5 presents the association between SP-D, IL-18, CCL2/MCP-1, and the clinical status of COVID-19 patients. Among these three parameters, only IL-18 showed a positive correlation with the disease severity (R = 0.228; *p* = 0.025). Accordingly, IL-18 levels exhibited a distinct upward trend as COVID-19 severity increased (Table 6).The Kruskal–Wallis ANOVA rank test indicated significant differences in IL-18 concentrations across patient groups stratified by disease severity (H = 17.7; *p* < 0.001), whereas no such differences were observed for SP-D and CCL2/MCP-1 (Table 6; Figure 4 and Figure 5). Post hoc analysis further showed that IL-18 was markedly elevated in critically ill patients compared to those with moderate severity (915 vs. 565 pg/mL; *p* < 0.001) (Table 6; Figure 6). In hospitalized patients, both CCL2/MCP-1 and IL-18 showed significant positive correlations with the extent of pulmonary involvement (R = 0.278, *p* = 0.017; and R = 0.471, *p* < 0.001, respectively), with IL-18 demonstrating the strongest association (Table 5). CTSS values also varied significantly by disease severity (H = 53.78; *p* < 0.001). Scores in critical and severe cases were significantly higher than in moderate cases (16.5 vs. 2, *p* = 0.001; and 14.5 vs. 2, *p* = 0.048, respectively) (Table 7).

**Figure 7:**
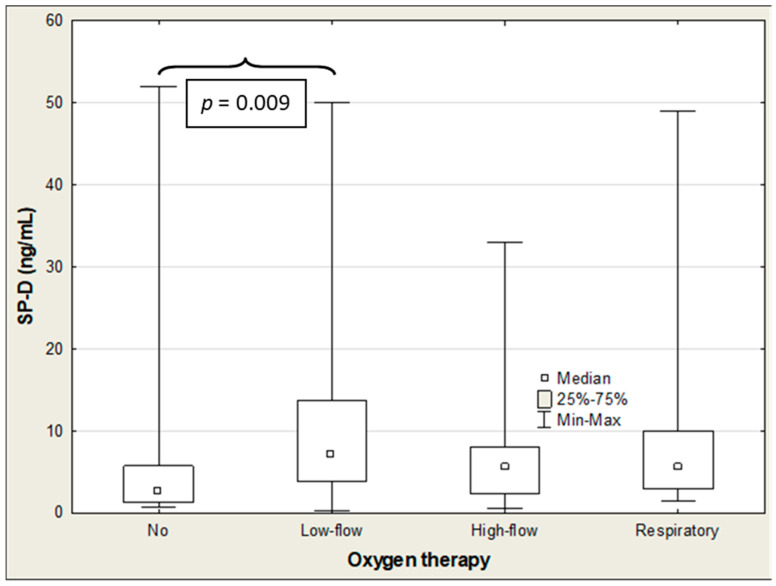
SP-D levels in COVID-19 patients receiving/not receiving oxygen therapy.

**Figure 8:**
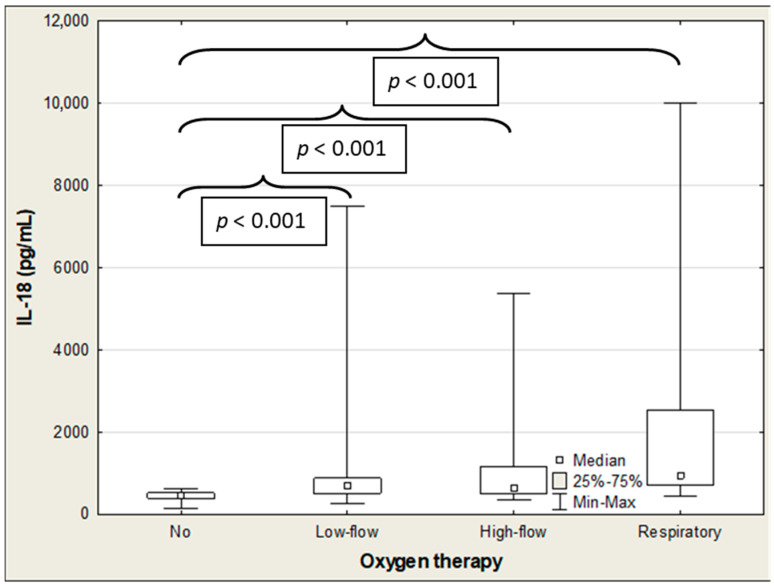
IL-18 concentrations in the COVID-19 patients with/without oxygen therapy.

**Figure 9:**
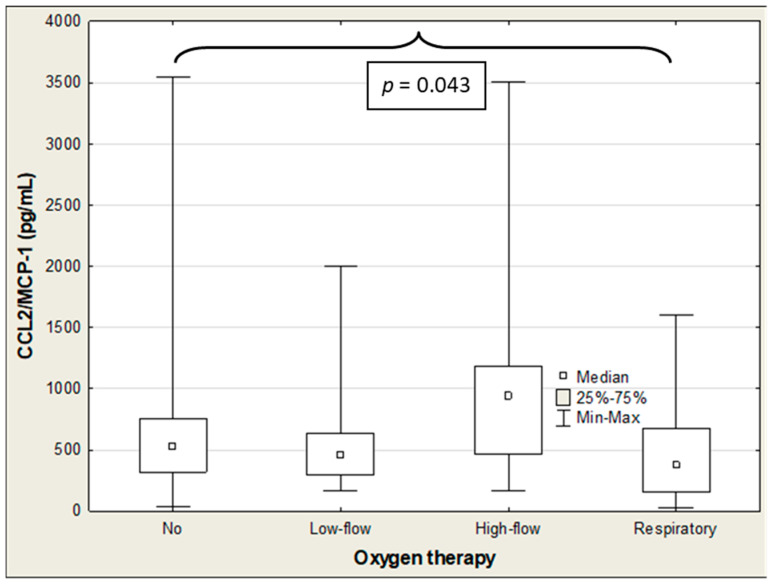
CCL2/MCP-1 levels in COVID-19 patients receiving/not receiving oxygen therapy.

The SP-D and IL-18 levels were significantly negatively correlated with oxygen therapy (R = −0.299 (*p* = 0.003) and R = −0.250 (*p* = 0.014), respectively), with SP-D showing the strongest association (Table 5). The ANOVA rank Kruskal–Wallis test revealed significant differences in the SP-D and IL-18 levels. These were determined based on the mode of oxygen administration (H = 10.2 (*p* = 0.017) and H = 25.6 (*p* < 0.001), respectively), and the difference in the CCL2/MCP-1 approached statistical significance (H = 7.3; *p* = 0.063) (Table 8). The median SP-D concentration was significantly higher in patients who needed low-flow oxygen therapy than in those who did not require oxygen administration (7.19 vs. 2.64; *p* = 0.009). The SP-D level decreased with the mode of oxygen administration, albeit the difference was not statistically significant (Table 8; Figure 7). The IL-18 concentration progressively increased with growing amounts of oxygen administration. There were significant differences between patients with oxygen therapy and those not requiring oxygen administration (low-flow therapy: 561 vs. 438 pg/mL (*p* < 0.001); high-flow therapy: 582 vs. 438 pg/mL (*p* < 0.001); and respiratory support: 760 vs. 438 (*p* < 0.001)) (Table 8; Figure 8). The median CCL2/MCP-1 levels were significantly higher in patients on mechanical ventilation than those without oxygen therapy (940 vs. 373 pg/mL; *p* = 0.043) (Table 8; Figure 9).

## 3. Discussion

This study aimed to evaluate the diagnostic usefulness of SP-D, CCL2/MCP-1, and IL-18 in patients with SARS-CoV-2 infection. This is visible particularly in the context of lung failure assessment and lung parenchyma involvement and the risk evaluation of developing pulmonary fibrosis at the onset of COVID-19.

It is known that most COVID-19 patients experience mild or moderate disease severity, but some of them become severely ill. Although some patients show improvement over months, others develop variable degrees of health sequelae. These include pulmonary, neurological, psychological, and cardiovascular complications. A small subset of patients, particularly those with severe or critical illness, experience pulmonary complications such as severe pneumonia and lung edema. These can progress to acute respiratory distress syndrome (ARDS) or multi-organ failure, both linked to unfavorable outcomes and elevated mortality rates [4]. Post-COVID-19 pulmonary fibrosis ranks among the most severe long-term sequelae, as noted by numerous studies. Meanwhile, short-term instances remain uncommon [6,23]. This pathological process develops especially in patients with severe pneumonia and pulmonary failure or ARDS, or in those requiring intensive respiratory support, such as mechanical ventilation. It is estimated that about 40% of patients with COVID-19 develop ARDS, 20% of which are severe [24]. There are many causes of pulmonary fibrosis. SARS-CoV-2 viral infection may be a cofactor for its pathogenesis. Hence, it is critical to identify patients who may develop acute respiratory failure and lung damage, and further pulmonary fibrosis, early.

Thus, it is necessary to search for tests that could identify lung injury due to COVID-19 in the early stage of the disease and could be predictors of pulmonary fibrosis. Currently, several laboratory tests are used to diagnose acute lung injuries and ARDS, as well as fibrosis-like changes. These are of particular importance in severe cases of COVID-19 and post-COVID-19 [4,11,12,15,17,25]. The parameters we studied are directly or indirectly related to lung tissue. Any changes in their serum concentrations may correspond to alterations in the respiratory system. A key question remains whether such changes can be measured to assess lung function and the degree of lung damage and, in the long-term, predict pulmonary fibrosis. Whether these tests could identify lung injury caused by COVID-19 at the beginning of the disease and be predictors of some complications, such as pulmonary fibrosis remains insufficiently evaluated. The early phase of SARS-CoV-2 infection is associated with the development of ground-glass opacity lung lesions. They are typical of interstitial pneumonia in a significant proportion of patients [26]. As such, the chronic coexistence of inflammation and fibrotic processes and possible gradual progression to pulmonary fibrosis is a hallmark of diseases featuring interstitial changes in the lungs [27]. Each parameter was also examined for its usefulness in assessing lung parenchyma involvement, analyzed using the computed tomography severity score (CTSS).

The COVID-19 patients included both females and males, aged 22–89 years, who were newly diagnosed and untreated upon hospital admission. They exhibited a range of disease severities, with symptom durations from 1 day to 3 weeks. At this stage of the disease, nearly 80% of patients had respiratory dysfunction and required oxygen administration, of which more than half needed low-flow therapy. Some of them required high-flow oxygen therapy or mechanical ventilation. One-third of patients were in the acute and critical phase of the disease, with a high degree of lung involvement measured with the CT severity score (CTSS). Most patients were not vaccinated and had chronic comorbidities.

The first biomarker we studied was SP-D, a pulmonary surfactant protein found in the alveolar space, also known as a lung-resident protein. Alterations in pulmonary surfactants may contribute to the pathogenesis of lung diseases such as ARDS and interstitial lung diseases. The serum SP-D concentration in COVID-19 patients was significantly increased before and after hospitalization compared with healthy subjects and did not change after treatment. Its concentration tended to increase with the disease severity evaluated with the MEWS. It was noted to be higher in critically ill patients compared to those in the moderate and acute phases. We showed a significant increase in SP-D levels in patients requiring oxygen therapy, already at the early stage of treatment with low-flow oxygen. This therapy is commonly used for patients with mild–moderate hypoxemia. The SP-D concentration was significantly negatively correlated with oxygen therapy. We observed that the serum SP-D level gradually increased with decreasing oxygen saturation and partial pressure of arterial oxygen. This shows a close relationship between SP-D and respiratory function; therefore, SP-D can be an indicator of the degree of respiratory lung function. Our results are consistent with existing literature. Certain studies report markedly elevated serum SP-D concentrations in COVID-19 patients at admission versus healthy controls in the infection’s initial stage. However, unlike in our study, these levels dropped 7 days after hospitalization [28]. Levels were higher in severe cases than in mild ones, according to disease severity [28]. The authors propose SP-D as a suitable marker for COVID-19 pneumonia, with early detection of its levels potentially vital for preventive clinical management. Several studies have shown that SP-D can be a predictive marker of COVID-19 and its outcome. The early detection of SP-D concentrations and their follow-up in hospitalized patients should be considered to direct therapeutic interventions [29,30,31]. Recently, it was shown that the SP-D level significantly increased in both patients with ARDS and those with fibrosis-like changes on CT scans. This suggests that most patients with ARDS induced by COVID-19 have a higher risk of pulmonary fibrosis than patients with ARDS [17]. Our findings indicate that SP-D is associated with oxygenation parameters and oxygen therapy requirements, supporting its role as a marker of respiratory failure and alveolar injury. Conversely, the lack of correlation with lung involvement and fibrosis-related markers suggests that SP-D is not a reliable predictor of pulmonary fibrosis.

A subsequent biomarker, CCL2/MCP-1, is a small cytokine, primarily secreted by monocytes, macrophages, and dendritic cells [18,32,33]. In general, CCL2 tightly regulates cellular processes and thereby recruits these immune cells to the sites of inflammation (especially to the lungs). This is produced by either tissue injury or infection, including SARS-COV-2 viral infection [18]. Excessive infiltration of immune cells and cytokine production in the lungs result in significant alveolar damage and respiratory failure. Several studies have reported upregulation of chemokine CCL2 in COVID-19 patients [34,35,36,37]. Consistent with our findings for SP-D, CCL2/MCP-1 levels were also elevated in all COVID-19 patients both before and after hospitalization compared to healthy individuals. Their concentration tended to increase with the disease severity. It was higher in severe and critically ill patients compared with those in the moderate phase. Regarding the amount and rate of oxygen administration, in contrast with SP-D, the CCL2/MCP-1 level did not correlate with oxygen therapy; however, its level was significantly higher only in patients requiring mechanical ventilation. CCL2/MCP-1 seems to be a less sensitive indicator of lung function than SP-D. Nevertheless, we observed that the CCL2/MCP-1 level in the serum gradually increased with decreasing partial pressure of arterial oxygen, showing a noteworthy correlation. In contrast with SP-D, we observed a substantial correlation between the CCL2/MCP-1 concentration and the pulmonary involvement severity as well as hyaluronic acid, which is a marker of fibrosis. CCL2/MCP-1 plays a pivotal role in both the acute inflammatory phase and the development of post-COVID-19 pulmonary fibrosis. This is evident in fostering monocyte/macrophage infiltration, stimulating fibroblast collagen synthesis, and exacerbating lung tissue damage [38]. This mechanism may lead to fibroproliferative disorders and pulmonary fibrosis after COVID-19-related acute respiratory distress syndrome (ARDS) [34,35,36,37,38]. Overall, SARS-CoV-2 infection disrupts normal lung tissue homeostasis and elevates CCL2/MCP-1 concentrations. Through this it enhances the recruitment and activation of inflammatory and profibrotic cells, which leads to lung injury, fibroblast proliferation, and excessive collagen deposition, characteristic of pulmonary fibrosis [34,35,36,37,38]. Hence, our results show that CCL2/MCP-1 may be an indicator of lung function and lung parenchyma involvement diagnosed via CTSS at the beginning of COVID-19, as well as a predictor of the development of pulmonary fibrosis. Furthermore, imaging studies, such as computed tomography, showed characteristic inflammatory changes. Nevertheless, abnormalities in the lung parenchyma can occur despite the absence of clinical signs of pneumonia.

IL-18 is a cytokine produced mainly by monocytes/macrophages in response to harmful stimuli such as viral infections [19]. Unlike the two previous tests, SP-D and CCL2/MCP-1, IL-18 levels were significantly higher in COVID-19 patients before hospitalization but decreased after therapy. The IL-18 concentration showed a higher amplitude of changes and responded faster to treatment. Furthermore, it is the only test that significantly correlated with disease severity, reaching the highest values in critical-stage patients compared with moderate-stage patients. Our research results are similar to those in the literature [20,21,22,39]. Moreover, IL-18 and oxygen therapy were significantly correlated; the IL-18 concentration increased with oxygen deficiency and was highest in patients requiring mechanical ventilation. This was evidenced by the negative correlation between IL-18 and oxygen saturation. Similar to CCL2/MCP-1, we observed a significant correlation between IL-18 and the degree of lung involvement and some markers of fibrosis. Some authors report that IL-18 levels are significantly elevated and correlate with severe disease in COVID-19 patients. This contributes to inflammatory lung injury and, potentially, to the development of pulmonary fibrosis as a post-COVID-19 complication [20,21,22]. Excessive IL-18 production can result in severe pathological injury by sustaining and amplifying the inflammatory response, leading to cytokine storm and tissue damage. This inflammation and immune dysregulation contribute to lung injury, which is a precursor to fibrosis development. In our study, IL-18 levels were significantly higher in patients with cytokine storm and were correlated with IL-6. IL-18 contributes to pulmonary fibrosis by driving excessive inflammasome activation, inflammatory cytokine release, and neutrophil-mediated lung injury [39]. These combined effects lead to tissue damage and fibrotic remodeling in the lungs of affected patients [20]. Recent studies have highlighted the role the cGAS-STING pathway in driving the NLRP3 inflammasome activation and subsequent IL-18 release during viral infections [40]. Increased IL-18 in COVID-19 reflects inflammasome-driven inflammation that may be facilitated by aberrant STING signaling. However, it should not be interpreted as a specific readout of STING-pathway activation alone. An important question is whether pharmacological modulation of the cGAS–STING axis, for example through agents such as fluvoxamine, could mitigate both the hyperinflammatory response observed during acute disease and the longer-term risk of fibrotic sequelae. Based on our results, IL-18 can be an indicator of respiratory failure and lung damage in the early stage of COVID-19 and could be a predictor of the development of pulmonary fibrosis.

The main limitations of this study are as follows. Firstly, it had a small sample size since only 66 of 86 patients were evaluated for lung involvement. The remaining patients had abnormalities in imaging studies that prevented a clear assessment (pneumothorax, pleural effusion, non-specific lesions, and overlapping lesions of a different nature); therefore, a CTSS assessment was not performed. Of the 66 patients, solely 10 were in the acute phase of the disease with acute pneumonia/pre-ARDS, and 12 were in the critical stage with pneumonia/ARDS. This could initiate fibrotic processes with the progressive development of pulmonary fibrosis. Secondly, the disease has a short duration. Blood samples were collected from patients only at the time of hospital admission, and their mean duration of COVID-19 symptoms was solely 5.8 days (range: 1–21 days). Comparing our data with those of other authors, in a lung computed tomography study involving a group of 114 patients with severe COVID-19, 6 months after the onset of the disease, 35% of patients showed pulmonary fibrosis-type changes, and 27% showed persistent ground-glass lesions and interstitial lung edema [41]. Similar results were obtained in a group of 171 patients, where fibrotic lung lesions were detected in chest CT scans in 19% of patients, 4 months after discharge from the hospital, including 39% of survivors who developed ARDS [42]. However, a systematic review of the literature data, including histopathological examinations, showed that fibrosis processes may begin as early as the third week of illness [43]. Thirdly, the effects of medications on the analyzed laboratory parameters were not assessed, mainly due to the small sample sizes of the respective treatment subgroups. Fourthly, the study is limited by the use of univariate analyses without multivariate modeling, although multiple variables—such as age, symptom duration, comorbidities, and vaccination status—may influence biomarker levels. Our research appears to be among the first to identify markers that are both indicators of respiratory failure and lesions in lung tissue, as well as potential predictors of pulmonary fibrosis in the early phase of COVID-19.

## 4. Materials and Methods

### 4.1. Patients

**Figure 10:**
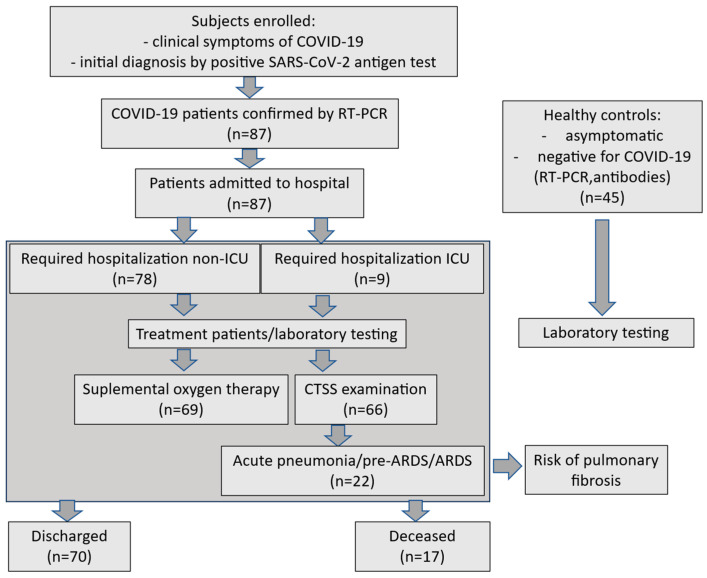
The flow chart for the recruitment of patients in this study.

The study included 87 COVID-19 patients (35 females, 52 males; mean age: 59.2 years, range: 22–89) admitted to the Department of Gastroenterology, Hepatology, and Internal Diseases with the Centre for Diagnostics and Endoscopic Treatment from 20 November 2021 to 16 March 2022. Symptomatic patients were initially screened for SARS-CoV-2 antigen detection with a Panbio^PM^ COVID-19 Ag Rapid Test Device (Abbott Rapid Diagnostics, Jena GmbH, Jena, Germany) in nasopharyngeal swabs, confirmed via RT-PCR assay for SARS-CoV-2 RNA (SARS-CoV-2 Triplex kits, Astra Biotech, Berlin, Germany; Azure Cielo 6 thermocycler, Azure Biosystems, Dublin, OH, USA). All patients underwent standard laboratory tests (biochemical, hematological, coagulometric, arterial blood gas, and CO-oximetry) plus imaging upon admission. The time from the onset of the first symptoms to sample collection varied and averaged 5.8 days (range: 1–21 days). The categorization of symptoms duration among the participants was as follows: 1–5 days: 37 subjects, 6–10 days: 27 individuals, 11–15 days: 6 patients, and 21–25 days: 1 case. Necessary laboratory examinations were conducted as needed throughout their hospital stay, based on clinical status, up to discharge or death. The patient recruitment flowchart appears in Figure 10.

**Figure 11:**
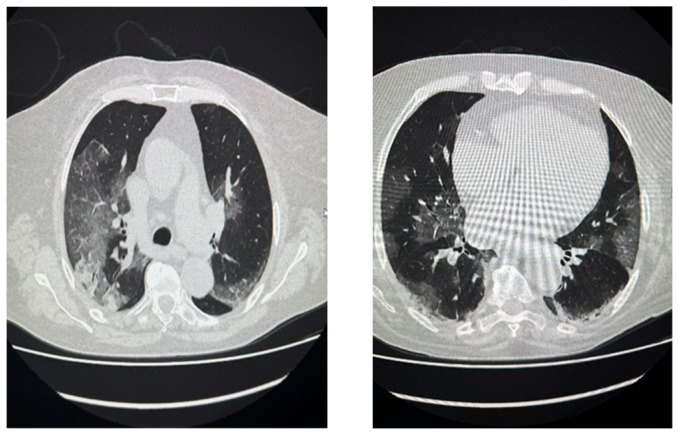
High-resolution computed tomography (HR-CT) of the chest. In both lungs, patchy areas of interstitial opacities of ground-glass appearance, with features of consolidation at the base of the right lung. In both lungs, scar-like fibrotic changes with thickening of the interlobular septa and features of bronchial traction. The appearance is typical in the course of COVID-19 infection (patient: 68-year-old female, day 9 of SARS-CoV-2 infection).

Patients were divided into three groups based on COVID-19 severity: the moderate group (58 patients), the severe group (13 patients), and the critical group (16 patients). The moderate group consisted of symptomatic individuals (such as those with fever, cough, and fatigue) showing lower respiratory tract involvement, but no respiratory failure, with pulse oximetry oxygen saturation (SatO2) ≥ 94% and only mild radiographic abnormalities. Severe COVID-19 patients had SatO2 < 94% alongside a respiratory rate >30 breaths/min, or lung infiltrates exceeding 50%, or a PaO2/FiO2 ratio < 300 with respiratory failure/or-ARDS. Critical cases involved multi-organ dysfunction with respiratory failure/ARDS [44]. Disease severity was assessed using the modified early warning score (MEWS), which incorporates five factors: systolic blood pressure, heart rate, respiratory rate, body temperature, and AVPU score (alert, verbal, pain, and unconscious) [45]. Each factor scored 0–3 points, yielding a total MEWS from 0 to 14. Moderate, severe, and critical patients scored < 3, 3–4, and >4 points, respectively. The chest computed tomography severity score (CTSS) quantified lung involvement in COVID-19 with some triage and prognostication value, as in [46]. This 25-point scale is based on the detection of lesions within each of the five lung lobes. The severity of lesions in each region is graded on a scale of 0–5 (0: no lesions; 1: <5% involvement; 2: 5–25% involvement; 3: 26–49% involvement; 4: 50–75% involvement; and 5: >75% involvement) [47,48]. The average baseline CTSS for COVID-19 patients was 7.9 (ranging from 0 to 24). Characteristic “ground-glass” opacities were observed in COVID-19 patients—white, patchy, hazy areas in the lungs, particularly in the lower lobes and peripheral regions [Figure 11}. Typical CTSS-associated lung alterations by disease stage at a total score of 7.9 points (moderate stage) corresponding to the progressive stage of disease (CTSS 6–15), included expanding ground-glass opacities, “crazy paving”, and early consolidation. In the severe-critical COVID-19 stage, diffuse bilaterial involvement, dense consolidation, and, in extreme cases, a “white lung”, and linear opacities/fibrotic-like changes appearance were observed. Patients were grouped by disease severity as follows: mild (≤8), moderate (from 9 to below 15), and severe (15–25). Only 66 of 87 patients were evaluated for lung involvement. The threshold for identifying patients with acute pneumonia in CTSS was 14.5 for severe patients and 16.5 for critical patients. Out of the total, 69 patients (79.3%) needed supplemental oxygen. The majority—39 cases (44.8%)—required low-flow nasal cannula oxygen therapy (oxygen flow up to 18 L/min at 25–35% concentration). Additionally, 21 patients (24.1%) received high-flow nasal cannula oxygen therapy (20–60 L/min flow, with 40–100% concentration adjusted based on oxygen saturation), and 9 (10.3%) required respiratory support. In the moderate severity group of 58 patients (66.7%), 18 (31%) needed no oxygen, 34 (58.6%) used low-flow therapy, and 6 (10.3%) got high-flow. Among severe cases, as many as 10 (76.9%) patients required high-flow, while only 3 (23.1%) needed low-flow oxygen therapy. For critical COVID-19 patients, 8 (57.1%) were placed on mechanical ventilation, 4 (28.6%) received high-flow, and 2 (14.3%) used low-flow oxygen therapy. Apart from the oxygen therapy, the patients underwent antiviral therapy (remdesivir—11 subjects), immunotherapy (tocilizumab—20 patients, baricitinib—3 patients) and the steroid treatment (dexamethasone—37 patients) until improvement or death.

Among patients with COVID-19, 28 (32.2%) had a cytokine storm, and 66 had chronic comorbidities (75.9%). Cytokine storm was identified by interleukin-6 (IL-6) levels exceeding 100 µg/mL. The most common chronic comorbidities included hypertension (21 patients), type 2 diabetes mellitus (15 patients), and cirrhosis (8 patients). Patient reports indicated that 29 individuals (33.3%) had received vaccination, while 55 had not. Among the vaccinated group, 9 had one dose, 12 had two doses, and 8 had three doses. This study involved 87 COVID-19 patients, of whom 17 died in the hospital (the mortality rate of 19.5%), 9 were transferred to the intensive care unit (ICU) (10.3%), and 70 were discharged (80.5%).

The control group consisted of 45 healthy volunteers (27 women and 18 men), aged 22–60 years (mean age: 31.7 years). Participants were asymptomatic, tested negative for SARS-CoV-2 via RT-PCR genetic testing, and lacked anti-SARS-CoV-2 antibodies. The laboratory characteristics for the study group and healthy controls are presented in Table 1. Written informed consent was obtained from patients and healthy subjects after explaining the nature of this study. This study was approved by the local research ethics committee of the Medical University of Bialystok (APK.002.107.2023).

### 4.2. Materials

Blood samples were collected from a peripheral vein on two occasions: upon admission, prior to any treatment (Sample 1), and following 9 days of hospitalization (Sample 2). The blood was allowed to clot fully at room temperature. The sera were then separated by centrifugation at 1500× *g* for 5 min, collected, and frozen at −80 °C pending analysis. Arterial blood for blood gas analysis and CO-oximetry was obtained using standard heparinized tubes. Besides serum, a portion of each blood sample was drawn into EDTA-2 tubes for hematological evaluation. For coagulation assays, samples were taken into tubes with 3.2% sodium citrate (citrated plasma).

### 4.3. Methods

#### 4.3.1. Determination of SP-D, IL-18, and CCL2/MCP-1

The SP-D (expected range: 0.935–18.9 ng/mL), IL-18 (expected range: 84.8–358 pg/mL), and CCL2/MCP-1 concentrations (expected range: 200–722 pg/mL) were measured separately via the quantitative sandwich enzyme immunoassay technique using the Quantikine ELISA Human SP-D Immunoassay Kit, the Quantikine ELISA Human Total IL-18/IL-1F4 Immunoassay Kit, and the Quantikine ELISA Human CCL2/MCP-1 Immunoassay Kit, respectively (R&D Systems, Minneapolis, MN, USA). In this method, monoclonal antibodies specific to human SP-D, IL-18, or CCL2/MCP-1 were pre-coated onto a microplate. Standards and samples were pipetted into the wells and incubated for 3 (SP-D) or 2 h (IL-18 and CCL2/MCP-1), and any SP-D, IL-18, and CCL2/MCP-1 present was bound by the immobilized antibodies. After washing away any unbound substances, the appropriate antibodies (a monoclonal antibody specific for human SP-D linked with an enzyme, horseradish peroxidase; a monoclonal antibody specific for human total IL-18 conjugated to biotin; and a polyclonal antibody specific for human CCL2/MCP-1 conjugated to horseradish peroxidase) were added to the wells and incubated for 2 (SP-D and CCL2/MCP-1) or 1 h (IL-18). Next, following a wash to remove any unbound antibody–enzyme reagent, a substrate solution (tetramethylbenzidine (TMB)) was added to the wells and incubated. Color developed in proportion to the amount of SP-D or CCL2/MCP-1 bound in the initial step. In the case of the IL-18 assay, following a wash to remove any unbound antibody–biotin reagent, an enzyme (horseradish peroxidase)-linked streptavidin was added to the wells. After washing away any unbound streptavidin–enzyme reagent, a substrate solution (tetramethylbenzidine (TMB)) was added to the wells and incubated for 30 min. Color developed in proportion to the amount of IL-18 bound in the initial step. For all tests, the color development was stopped, and the intensity of the color (optical density) was measured using the Anthos 2020 microplate reader set at 450 nm (Instruments GmbH, Wals, Austria). The SP-D, IL-18, and CCL2/MCP-1 concentrations were read from the standard curve. The linearity of the assays extended up to 40 ng/mL for SP-D, up to 1000 pg/mL for IL-18, and up to 2000 pg/mL for CCL2/MCP-1 (samples with higher concentrations of tests were diluted with the appropriate calibrator diluent, and levels were read from the standard curve and multiplied by the dilution factor). The minimum detectable dose (MDD) of SP-D ranged from 0.02 to 0.037 ng/mL (the mean MDD was 0.11 ng/mL), and the total precision (coefficient of variance (CV)) was 6.2% at 5.99 ng/mL, 8.2% at 11.3 ng/mL, and 6.7% at 24.3 ng/mL. Moreover, the minimum detectable dose (MDD) of IL-18 ranged from 0.296 to 5.15 pg/mL (the mean MDD was 1.25 pg/mL). The intra-assay precision (coefficient of variance (CV)) was 3.1% at 126 pg/mL, 2.5% at 251 pg/mL, and 2.9% at 495 pg/mL. The minimum detectable dose (MDD) of CCL2/MCP-1 ranged from 0.57 to 10.0 pg/mL (the mean MDD was 1.7 pg/mL). The intra-assay precision (coefficient of variance (CV)) was 7.8% at 76.7 pg/mL, 4.7% at 364 pg/mL, and 4.9% at 1121 pg/mL.

#### 4.3.2. Biochemical Measurements

Serum activity of alanine aminotransferase (ALT; reference ranges: 5–41 IU/L for men, 5–33 IU/L for women), aspartate aminotransferase (AST; 5–36 IU/L), γ-glutamyltransferase (GGT; 10–71 IU/L for men, 6–42 IU/L for women), lactate dehydrogenase (LDH; 135–225 IU/L for men, 135–214 IU/L for women), and serum levels of C-reactive protein (CRP; <5 mg/L), and ferritin (30–400 µg/L for men, 15–150 µg/L for women) were determined using routine laboratory methods with a COBAS c501 analyzer (Roche/Hitachi, Tokyo, Japan). Interleukin 6 (IL-6; <7 pg/mL) was assessed by electrochemiluminescence assay on a COBAS e-411 (Roche Diagnostics, Rotkreuz, Switzerland).

#### 4.3.3. Hematological Assays

The complete blood count (CBC) analysis was performed using a Sysmex XN-1000 analyzer (Sysmex Corporation, Singapore). The reference ranges applied were as follows: RBC: 4.2–5.4 × 10^6^/µL (men) and 3.5–5.2 × 10^6^/µL (women); Hb: 14–18 g/dL (men) and 12–16 g/dL (women); Ht: 40–54% (men) and 37–47% (women); MCV: 82–93 fL; WBC: 4–10 × 10^3^/µL; PLT: 125–400 × 10^3^/µL.

#### 4.3.4. Coagulometric Test

Fibrinogen (reference range: 180–400 mg/dL) was conducted using the ACL TOP 300 CTS analyzer (Instrumentation Laboratory, Werfen Company, Bedford, MA, USA).

#### 4.3.5. Arterial Blood Gas (ABG) and CO-Oximetry

Arterial blood gas and CO-oximetry were conducted using an ABL 90 FLEX PLUS analyzer (Radiometer Medical ApS, Brønshøj, Denmark). The reference ranges for these studies were as follows: pH: 7.35–7.45; PaO_2_: 75–100 mmHg; PaCO_2_: 32–45 mmHg; base excess (BE): −2.5 to +2.5; and oxygen saturation (O_2_Sat): 95–98%.

### 4.4. Statistics

Statistical analysis was conducted with Statistica 13.3 PL software (StatSoft, Kraków, Poland). The results are presented as medians with interquartile ranges (Q1–Q3). Data normality was assessed via the Kolmogorov–Smirnov test. This showed normal distributions for Hb, RBCs, MCV, PLTs, and PaO2. For these normally distributed variables, the differences between Sample 1 and the controls, as well as Sample 2 and the controls, were evaluated using Student’s *t*-test. The Mann–Whitney U test analyzed the other variables with a non-normal distribution. The comparisons between Samples 1 and 2 employed the Wilcoxon matched-pairs test. The effects of the disease severity, oxygen therapy, and modified early warning score (MEWS) on SP-D, IL-18, and CCL2/MCP-1 levels were examined with the ANOVA rank Kruskal–Wallis test, followed by post hoc analysis. Spearman’s rank correlation coefficient was used for tests with a non-normal distribution, while Pearson’s correlation test was used for tests with a normal distribution. The results were considered statistically significant when *p*-values were less than 0.05.

## 5. Conclusions

In conclusion, SP-D, CCL2/MCP-1, and IL-18 may be indicators of respiratory function at the beginning of COVID-19 infection. SP-D may serve as a marker of alveolar injury. CCL2/MCP-1 and IL-18 may also reflect pulmonary parenchymal involvement and could be potential predictors of the risk of developing pulmonary fibrosis during COVID-19.

## Figures and Tables

**Table 1:** Laboratory characteristics of the study and the control groups.

Test	Sample 1 (S1)	Sample 2 (S2)	Controls (C)	Comparisons (*p*-Value)		
				S1 vs. C	S2 vs. C	S1 vs. S2
Biochemical Tests						
ALT (IU/L)	30.7 15.25–56.0	35.0 22.0–62.0	10.1 7.75–14.6	<0.001 *	<0.001 *	0.074
AST (IU/L)	42.0 23.75–71.0	33.3 20.15–50.35	19.85 17.15–23.3	<0.001 *	<0.001 *	0.005 *
GGT (IU/L)	52.0 26.0–120	75.0 31.0–150	14.0 10.0–20.0	<0.001 *	<0.001 *	0.865
LDH (IU/L)	268 204–399	182.0 161.0–253.5	155.0 139.0–180.5	<0.001 *	<0.001 *	<0.001 *
Total bilirubin (mg/dL)	0.52 0.35–0.73	0.45 0.25–0.88	0.47 0.35–0.58	0.273	0.846	0.229
Inflammatory Markers						
IL-6 (pg/mL)	42.0 19.4–127	24.6 12.8–65.9	1.50 1.50–1.82	<0.001 *	<0.001 *	0.253
CRP (mg/L)	41.1 18.1–109.1	12.2 4.00–36.9	0.64 0.60–1.19	<0.001 *	<0.001 *	<0.001 *
Procalcitonin (ng/mL)	0.13 0.06–0.44	0.080 0.044–0.36	0.027 0.021–0.033	<0.001 *	<0.001 *	0.130
Ferritin (µg/L)	593.5 236–1414	671.7 303.7–1245	54.5 27.3–114.5	<0.001 *	<0.001 *	0.005 *
Fibrinogen (mg/dL)	431 308–613	315.5 179.5–413	294 256–342	<0.001 *	0.722	0.465
Hematological Parameters						
Hb (g/dL)	12.9 11.2–14.0	12.6 11.0–13.8	13.7 13.2–14.8	<0.001 *	<0.001 *	0.895
Ht (%)	38.4 33.4–41.3	37.6 33.5–40.8	39.1 37.1–42.35	0.028 *	0.010 *	0.570
RBCs (×10^6^/µL)	4.27 3.68–4.65	4.13 3.75–4.61	4.59 4.32–4.93	<0.001 *	<0.001 *	0.895
MCV (fL)	89.2 85.5–94	89.5 85.7–93.25	85.6 83.5–88.4	0.001 *	<0.001 *	0.041 *
WBC (×10^3^/µL)	7.2 4.85–9.20	6.95 5.18–9.90	6.49 5.52–7.26	0.202	0.201	0.047 *
PLTs (×10^3^/µL)	205.5 163–270	254.0 158–345	247.5 213.5–271	0.087	0.463	0.002 *
Arterial Blood Gas and CO-Oximetry						
pH	7.457 7.426–7.484	7.459 7.436–7.488	7.405 7.375–7.425	<0.001 *	<0.001 *	0.468
PaO_2_ (mmHg)	83.6 64.0–107	82.6 65.7–107	97.0 93.0–101	0.008 *	0.007 *	0.317
PaCO_2_ (mmHg)	36.1 34.0–39.8	38.5 34.9–41.3	39.0 37.0–41.0	0.002 *	0.241	0.058
O_2_Sat (%)	96.8 93.0–98.5	96.7 94.9–98.1	97.0 97.0–99.0	0.204	0.075	0.839
BE (mEq/L)	2.50 −0.70–4.90	3.25 1.20–5.30	0.33 −0.90–1.15	0.002 *	<0.001 *	0.020 *

Data are reported as medians with interquartile ranges (Q1–Q3). Differences between S1 and C, as well as S2 and C, were assessed via Student’s *t*-test (for Hb, RBCs, MCV, PLTs, and pO_2_) or Mann–Whitney U test (for all other parameters). Comparisons between S1 and S2 were performed using the Wilcoxon matched-pairs test. * *p* < 0.05 indicates statistical significance. Abbreviations: ALT, alanine aminotransferase; AST, aspartate aminotransferase; GGT, γ-glutamyltransferase; LDH, lactate dehydrogenase; IL-6, interleukin 6; CRP, C-reactive protein; Hb, hemoglobin; Ht, hematocrit; RBCs, red blood cells; MCV, mean corpuscular volume; WBC, white blood cells; PLTs, platelets; PaO_2_, partial pressure of arterial oxygen; PaCO_2_, partial pressure of arterial carbon dioxide; O_2_Sat, oxygen saturation; BE, base excess.

**Table 2:** Serum SP-D, IL-18, and CCL2/MCP-1 levels in the study group and the controls.

Test	Sample 1 (S1)	Sample 2 (S2)	Controls (C)	Comparisons (*p*-Value)		
				S1 vs. C	S2 vs. C	S1 vs. S2
SP-D (ng/mL)	5.73 2.33–9.52	6.53 3.72–14.0	3.04 1.73–4.20	<0.001 *	<0.001 *	0.510
IL-18 (pg/mL)	606 456–862	302 116–532	159 69–259	<0.001 *	0.027 *	<0.001 *
CCL2/MCP-1 (pg/mL)	504 295–755	553 351–827	262 230–364	<0.001 *	<0.001 *	0.657

Data are reported as medians with interquartile ranges (Q1–Q3). Differences between S1 and C, as well as S2 and C, were assessed via the Mann–Whitney U test. Differences between S1 and S2 were evaluated using the Wilcoxon matched-pairs test. * *p* < 0.05 denotes statistical significance. Abbreviations: SP-D, surfactant protein D; IL-18, interleukin-18; CCL2/MCP-1, CC chemokine ligand 2/monocyte chemoattractant protein-1.

**Table 3:** Serum SP-D, IL-18, and CCL2/MCP-1 levels in different groups of COVID-19 patients.

COVID-19 Patients	Clinical Characteristics		
	Yes	No	Comparisons *(p*-Value)
Cytokine storm			
SP-D	5.86 (3.01–8.1)	5.64 (2.31–9.64)	0.595
IL-18	590 (302–935)	517 (390–628)	<0.001
CCL2/MCP-1	571 (309–1027)	471 (268–667)	0.085
Comorbidities			
SP-D	5.97 (2.81–9.52)	4.29 (2.23–8.96)	0.437
IL-18	532 (354–783)	544 (425–688)	0.374
CCL2/MCP-1	530 (295–755)	483 (223–723)	0.718
Vaccination			
SP-D	6.36 (3.64–9.64)	4.97 (2.01–8.10)	0.082
IL-18	582 (336–760)	516 (354–758)	0.538
CCL2/MCP-1	504 (268–819	459 (295–722)	0.852
Surviving/non-surviving			
SP-D	5.78 (2.27–9.52)	5.38 (3.01–9.52)	0.623
IL-18	878 (651–1583)	578 (436–763)	<0.001
CCL2/MCP-1	555 (338–1027)	453 (268–695)	0.107

Data are presented as medians and interquartile ranges (Q1–Q3). The differences in SP-D levels between different groups of patients were estimated via the Mann–Whitney U test. Significant difference at *p* < 0.05. Abbreviation: SP-D, surfactant protein D; interleukin-18; CCL2/MCP-1, chemokine (CC-motif) ligand 2/monocyte chemoattractant protein-1.

**Table 4:** The association between SP-D, IL-18, CCL2/MCP-1, and laboratory tests in COVID-19 patients.

Test	SP-D		IL-18		CCL2/MCP-1	
	R	*p*	R	*p*	R	*p*
ALT (IU/L)	0.019	0.858	0.298	0.003	−0.033	0.745
AST (IU/L)	0.142	0.169	0.389	<0.001	0.082	0.429
GGT (IU/L)	0.066	0.529	0.242	0.019	−0.016	0.115
LDH (IU/L)	0.178	0.087	0.398	<0.001	0.153	0.142
Total bilirubin (mg/dL)	0.013	0.902	0.330	0.001	0.023	0.821
IL-6 (pg/mL)	0.146	0.162	0.508	<0.001	0.171	0.098
CRP (mg/L)	0.068	0.509	0.445	<0.001	0.143	0.164
Procalcitonin (ng/mL)	−0.076	0.468	0.483	<0.001	0.068	0.512
Ferritin (µg/L)	−0.007	0.946	0.544	<0.001	0.169	0.099
Galectin 3 (ng/mL)	0.053	0.616	0.547	<0.001	0.075	0.464
HA (ng/mL)	0.025	0.819	0.340	0.001	0.218	0.042
TNF-α	−0.027	0.797	0.269	0.008	0.100	0.333
IFN-γ	0.104	0.314	0.508	<0.001	0.265	0.009
WBCs (×10^3^/µL)	−0.051	0.626	0.326	0.001	0.012	0.905
Fibrinogen (mg/dL)	0.044	0.687	0.153	0.161	0.066	0.549
PaO_2_ (mmHg)	−0.240	0.026	−0.183	0.089	−0.219	0.042
PaCO_2_ (mmHg)	0.005	0.960	−0.093	0.376	0.073	0.488
O_2_Sat (%)	−0.206	0.048	−0.218	0.036	−0.151	0.149

R, Spearman’s rank correlation coefficient. *p* < 0.05: significant correlation. Abbreviations: ALT, alanine aminotransferase; AST, aspartate aminotransferase; GGT, γ-glutamyltransferase; LDH, lactate dehydrogenase; IL-6, interleukin-6; IL-18, interleukin-18; SP-D, surfactant protein D; CCL2/MCP-1, chemokine (CC-motif) ligand 2/monocyte chemoattractant protein-1; CRP, C-reactive protein; HA, hyaluronic acid; TNF-α, tumor necrosis factor alpha; IFN-γ, interferon gamma; WBCs, white blood cells; PaO_2_, partial pressure of arterial oxygen; PaCO_2_, partial pressure of arterial carbon dioxide; O_2_Sat, oxygen saturation.

**Table 5:** The association between SP-D, IL-18, CCL2/MCP-1, and clinical status in COVID-19 patients.

Test	SP-D		IL-18		CCL2/MCP-1	
	R	*p*	R	*p*	R	*p*
Disease severity	0.034	0.745	0.228	0.025	0.043	0.680
Pulmonary involvement severity	0.160	0.177	0.471	<0.001	0.278	0.017
Oxygen therapy	−0.299	0.003	−0.250	0.014	−0.053	0.607

R, Spearman’s rank correlation coefficient. *p* < 0.05: significant correlation. Abbreviations: SP-D, surfactant protein D; IL-18, interleukin-18; CCL2/MCP-1, chemokine (CC-motif) ligand 2/monocyte chemoattractant protein-1.

**Table 6:** Serum SP-D, IL-18, and CCL2/MCP-1 levels according to COVID-19 severity.

COVID-19 Severity	SP-D (ng/mL)	IL-18 (pg/mL)	CCL2/MCP-1 (pg/mL)
Moderate (n = 58)	5.73 2.23–9.64	565 430–751	465 286–710
Severe (n = 13)	5.51 3.31–8.25 ^1^ *p* = 1.000	652 456–883 ^1^ *p* = 0.517	554 244–1069 ^1^ *p* = 1.000
Critical (n = 16)	6.32 2.96–8.98 ^1^ *p* = 1.000 ^2^ *p* = 1.000	915 695–3981 ^1^ *p* < 0.001 * ^2^ *p* = 0.079	532 365–1061 ^1^ *p* < 0.611 ^2^ *p* = 1.000

Data are reported as medians with interquartile ranges (Q1–Q3). Levels of SP-D, IL-18, and CCL2/MCP-1 varied across disease severity groups. They were assessed using Kruskal–Wallis rank ANOVA tests: H = 0.268 (*p* = 0.874); H = 17.7 (*p* < 0.001); and H = 1.87 (*p* = 0.393), followed by post hoc analyses. * Significant at *p* < 0.05. ^1^ Compared to moderate stage. ^2^ Compared to severe stage. Abbreviations: SP-D, surfactant protein D; IL-18, interleukin-18; CCL2/MCP-1, chemokine (CC-motif) ligand 2/monocyte chemoattractant protein-1.

**Table 7:** CTSS score values in the COVID-19 patients according to disease severity.

COVID-19 Severity	CTSS
Moderate (n = 44)	2 0−10
Severe (n = 10)	14.5 11−15 ^1^ *p* = 0.048 *
Critical (n = 12)	16.5 10−20 ^1^ *p* = 0.001 *

Data are reported as medians with interquartile ranges (Q1–Q3). CTSS values varied across levels of disease severity, as shown by the Kruskal–Wallis ANOVA rank test (H = 53.78; *p* < 0.001), followed by post hoc analysis. * Significant difference at *p* < 0.05. ^1^ Compared to the moderate stage. Abbreviations: CTSS, chest computed tomography severity score.

**Table 8:** Serum SP-D, IL-18, and CCL2/MCP-1 levels in the COVID-19 patients according to oxygen therapy.

Oxygen Therapy	SP-D (ng/mL)	IL-18 (pg/mL)	CCL2/MCP-1 (pg/mL)
No (n = 18)	2.64 1.26–5.73	438 384–519	373 157–675
Low-flow oxygen (n = 39)	7.19 3.90–13.7 ^1^ *p* = 0.009	561 367–764 ^1^ *p* < 0.001 *	522 320–755 ^1^ *p* = 1.000
High-flow oxygen (n = 21)	5.64 2.31–8.0 ^1^ *p* = 0.488 ^2^ *p* = 1.000	582 354–830 ^1^ *p* < 0.001 ^2^ *p* = 1.000	453 296–638 ^1^ *p* = 1.000 ^2^ *p* = 1.000
Respiratory (n = 9)	5.95 2.91–9.97 ^1^ *p* = 0.459 ^2^ *p* = 1.000 ^3^ *p* = 1.000	760 302–958 ^1^ *p* < 0.001 ^2^ *p* = 0.298 ^3^ *p* = 0.732	940 465–1183 ^1^ *p* = 0.043 * ^2^ *p* = 0.326 ^3^ *p* = 0.292

Data are reported as medians with interquartile ranges (Q1–Q3). Levels of SP-D, IL-18, and CCL2/MCP-1 varied across oxygen administration categories. This was shown by Kruskal–Wallis ANOVA rank tests: H = 10.2 (*p* = 0.017); H = 25.6 (*p* < 0.001); H = 7.3 (*p* = 0.063); and H = 4.87 (*p* = 0.182), followed by post hoc analyses. * Significant at *p* < 0.05. ^1^ Compared to no oxygen therapy. ^2^ Compared to low-flow oxygen therapy. ^3^ Compared to high-flow oxygen therapy. Abbreviations: SP-D, surfactant protein D; interleukin-18; CCL2/MCP-1, chemokine (CC-motif) ligand 2/monocyte chemoattractant protein-1.

## Data Availability

The original contributions presented in this study are included in the article. Further inquiries can be directed to the corresponding author.
